# Supplementary material for: Characterization of a Novel N4-Methylcytosine Restriction-Modification System in Deinococcus radiodurans
Source: Int J Mol Sci. 2024 Jan 29;25(3):1660. doi: 10.3390/ijms25031660 (PMC10855626; doi:10.3390/ijms25031660)
Supplement: Supplementary file 1 [file ijms-25-01660-s001.zip › ijms-2811308-supplementary.pdf]

Supplementary Table S1. Strains and plasmids used in this study.

Strain and plasmid	Relevant feature	Reference or source
**Strains**		
D.radiodurans		
D. radiodurans R1	wild-type strain	ATCC13939
Δ*M.DraR1*	R1 but *M.DraR1::Str*	Li, S. J. *et al.*
Δ*R.DraR1*	R1 but *R.DraR1::kana*	This study
Δ*R*+Δ*M*	R1 but *M.DraR1::Str and R.DraR1::kana*	This study
E. coli		
DH(5α)	supE44, ΔlacU169 (φ80lacZΔM15), hsdR17, recA1, endA1, gyrA96, thi-1, relA1	TransGen Biotech, Beijing, China
ER2566	sulA11 Δ(mcrC-mrr)114::IS10 R(mcr-73::miniTn10-TetS)2 R(zgb-210::Tn10)(TetS) endA1 [dcm]	ZonHon Biopharma, Jiangshu, China.
ER2796	fhuA2 Δ (lacZ)r1 glnV44 trp-31 dcm-6 his-1 zed-501::Tn10 argG6 rpsL104 dam-16::Kan xyl-7 mtl-2 metR1 mcr-62 Δ (mcrB-hsd-mrr)114	Prof. Richard J. Roberts, NEB.
**Plasmid**		
pUC19	*E. Coli* vector	TransGen Biotech, Beijing, China
pRADK	*E. coli–D. radiodurans* shuttle vector, confers ampicillin resistance in *E. coli* and chloromycetin resistance in *D. radiodurans*	Laboratory stock
pRADKm	Modified pRADK vector contains one 'CCGCGG' site	Li, S. J. *et al.*
M. pRADKm	Methylated pRADKm vector with M.DraR1 enzyme	Li, S. J. *et al.*
pUC19-pDr-Chl (NS)	A strong promoter pDr and ΔCrtB fragment was inserted into BamHI and HindIII restriction sites	This study
pUC19-pDr-CCGCGG-Chl (S)	NS with an additional CCGCGG site just after the start codon	This study
pET28a-sumo	pET28 plasmid modified with a sumo tag and a Ulp1 protease site	Laboratory stock
pET28a-sumo-R.DraR1	pET28-sumo ligated with *R.DraR1*	This study
pUC19-pGroES-LacI-pDr-tetR	pGroES-LacI-pDr-tetR fragment was inserted into BamHI and HindIII restriction sites	This study
pRADKIS	Modified pRADK vector, pGroES-*LacI* was inserted into HindIII and XhoI, and pGroES was replaced by pSpac	This study
pRADKIS-LacZ	*LacZ* was inserted into NdeI and BamHI	This study
pRADKIS-R.DraR1	*R.DraR1* was inserted into NdeI and BamHI	This study

Supplementary Table S2. Primers or oligonucleotides used in this study.

**Primers for PCR amplification and sequencing**	**Sequence (5′→3′)**
R.DraR1_P1	TTTCCTGTGGTGGACTGGTTTG
R.DraR1_P2_K	GACGCGTCTGCAGAAGCTTgctgacaacaccagatgggg
K_F_P2	ccccatctggtgttgtcagcAAGCTTCTGCAGACGCGTC
R.DraR1_P3_K	TTTCATTTGATGCTCGATGAGTTTTTCTAGGccttccaagtctgtgagcg
K_R_P3	cgctcacagacttggaaggccTAGAAAAACTCATCGAGCATCAAATGAAA
R.DraR1_P4	CATCAACTTTCTGGTGCTTGGAG
R.DraR1_P5	ctacggctacctctttagcatgg
R.DraR1_P6	ggcaactgctaggcgctta
M.DraR1-P1	CACCCCCGTCCAGACTCAGC
M.DraR1-P4	GTGCCCATCTGGAGTCGCTACC
M.DraR1-P5	GTGAACTGGATTGCGGGATT
M.DraR1-P6	TTCCGCAGGTAGTGATAGTTGTTC
CrtB_P1	GGGCTTGGTCTGGTCGG
CrtB_R_P2_pDrChl	TCGTGGTGGCCTTTGACGGCCTGCTTACAGAAAAGGAAAGACA
pDrChl_F_P2_CrtB	TGTCTTTCCTTTTCTGTAAGCAGGCCGTCAAAGGCCACCACGA
CrtB_F_P3_Chl	CAGGGCGGGGCGTAATCTGTCACCCCTCCGGG
Chl_R_P3_CrtB	CCCGGAGGGGTGACAGATTACGCCCCGCCCTG
CrtB_P4	TGCCGGGTAGATTGCCG
pDrChl_R_CCGCGG	ACGGTGGTATATCCAGTGATTTTTTTCTCccgcggCATTTTAGCTTCCTTAGCTCCTGAAAATCT
Chl_F_CCGCGG	AGATTTTCAGGAGCTAAGGAAGCTAAAATGccgcggGAGAAAAAAATCACTGGATATACCACCGT
pDRChl_F_SOE	TGTCTTTCCTTTTCTGTAAGCAGGC
pDRChl_R_SOE	CCCGGAGGGGTGACAGA
28a_Sumo_FNde	CCGCGCGGCAGCCATATGAGCGATAGCGAAGTTAACCAA
28a_C10_F_sumo	TCGTGAGCAGATTGGTGGTagaggtgagctggggca
28a_Sumo_R_C10	tgccccagctcacctctACCACCAATCTGCTCACGA
28a_C10_RX	tggtggtggtgctcgagtcacttctcagatgcttcctcaat
AmyE_F_P1-NEW	GAGCAGAACAAGGTGCGTG
AmyEUP_FH-pUC19	catgattacgccaagcttGAGCAGAACAAGGTGCGTG
pGroESLacI_P2_FH-AmyEUP	GAACGCTAGCATCTCCCCCAAGCTTCTGCAGACGCGT
pGroESLacI_RB-tcR	GGGCACCAATAACTGCCTTAAAAAAAGGATCCTTACTGCCCGCTTT
pGroES_F_P2-AmyEUP	ACGCTAGCATCTCCCCCTGGAAGCACGTATTGTCGCC
pGroES_R-LacI	GACATCGTATAACGTTACTGGTTTCACGGGTCCTCCTGTGAGTG

	AGA
LacI_F-pGroES	TCTCACTCACAGGAGGACCCGTGAAACCAGTAACGTTATACGATGTC
LacI_R_P3-AmyEDown	GAGCAAGTCAGACTCCTCCTCTCACTGCCCGCTTTCCAG
tcR_RB-pGroESLacI	AAAGCGGGCAGTAAGGATCCTTTTTTTAAGGCAGTTATTGGTGCCC
TcR_F-pDr	GAGATTTTCAGGAGCTAAGGAAGCTAAAATGAAATCTAACAATGCGCTCATCGT
pDr_R-TcR	ACGATGAGCGCATTGTTAGATTTCATTTTAGCTTCCTTAGCTCCTGAAAATCTC
pDr_F_P3-AmyEDown	GGAGCAAGTCAGACTCCTCCTCCGTCAAAGGCCACCAC
AmyEDown_F_P3	GTGGTGGCCTTTGACGGAGGAGGAGTCTGACTTGCTCC
AmyE_R_P4_NEW	TGGACTTTGGCCTTCAGCG
pGroES_RX	AGCTCGCGAGGCCTCGAGTCACTGCCCGCTTTCCAG
pGroES_FH-pSpac	ctttccgctcttgtgctgttagAAGCTTTGGAAGCACGTATTGTCGCC
pSpac_FH-pGroES	GGCGACAATACGTGCTTCCAAAGCTTctaacagcacaagagcggaaag
pSpac_RBS_RN	TTGAATATGGCTCATATGTTGTCCTCCTGTaattgtGAGcgctcacaattcc
LacI-pGroES_FX_SOE	AAGCTCGCGAGGCCTC
pSpac_RN_SOE	TTGAATATGGCTCATATGTTGTCCTCC
LacZ_FN-pRADIS	ACAGGAGGACAACATatgcacggttacgatgcgc
LacZ_RB-pRADIS	GCAGGTCGAATCGGATCCttatttttgacaccagaccaactggtaatg
pRADIS_C10_FN	ACAGGAGGACAACATATGagaggtgagctggggca
pRADIS_C10_RB	GCAGGTCGAATCGGATCCtcacttctcagatgcttcctcaatcg
**Oligonucleotides**	
S30_1_F	AGACCCACGCCAtCGCGGTGGAGATTACGT
S30_1_R	ACGTAATCTCCACCGCGaTGGCGTGGGTCT
S30_2_F	AGACCCACGCCACtGCGGTGGAGATTACGT
S30_2_R	ACGTAATCTCCACCGCaGTGGCGTGGGTCT
S30_3_F	AGACCCACGCCACCtCGGTGGAGATTACGT
S30_3_R	ACGTAATCTCCACCGaGGTGGCGTGGGTCT
S30_4_F	AGACCCACGCCACCGtGGTGGAGATTACGT
S30_4_R	ACGTAATCTCCACCaCGGTGGCGTGGGTCT
S30_5_F	AGACCCACGCCACCGCtGTGGAGATTACGT
S30_5_R	ACGTAATCTCCACaGCGGTGGCGTGGGTCT
S30_6_F	AGACCCACGCCACCGCGtTGGAGATTACGT
S30_6_R	ACGTAATCTCCAaCGCGGTGGCGTGGGTCT
S30_F_3′FAM	AGACCCACGCCACCGCGGTGGAGATTACGT*(3′FAM)
S30_R	ACGTAATCTCCACCGCGGTGGCGTGGGTCT
S30_NS_F	AGACCCACGCCATATATATGGAGATTACGT
S30_NS_R	ACGTAATCTCCATATATATGGCGTGGGTCT

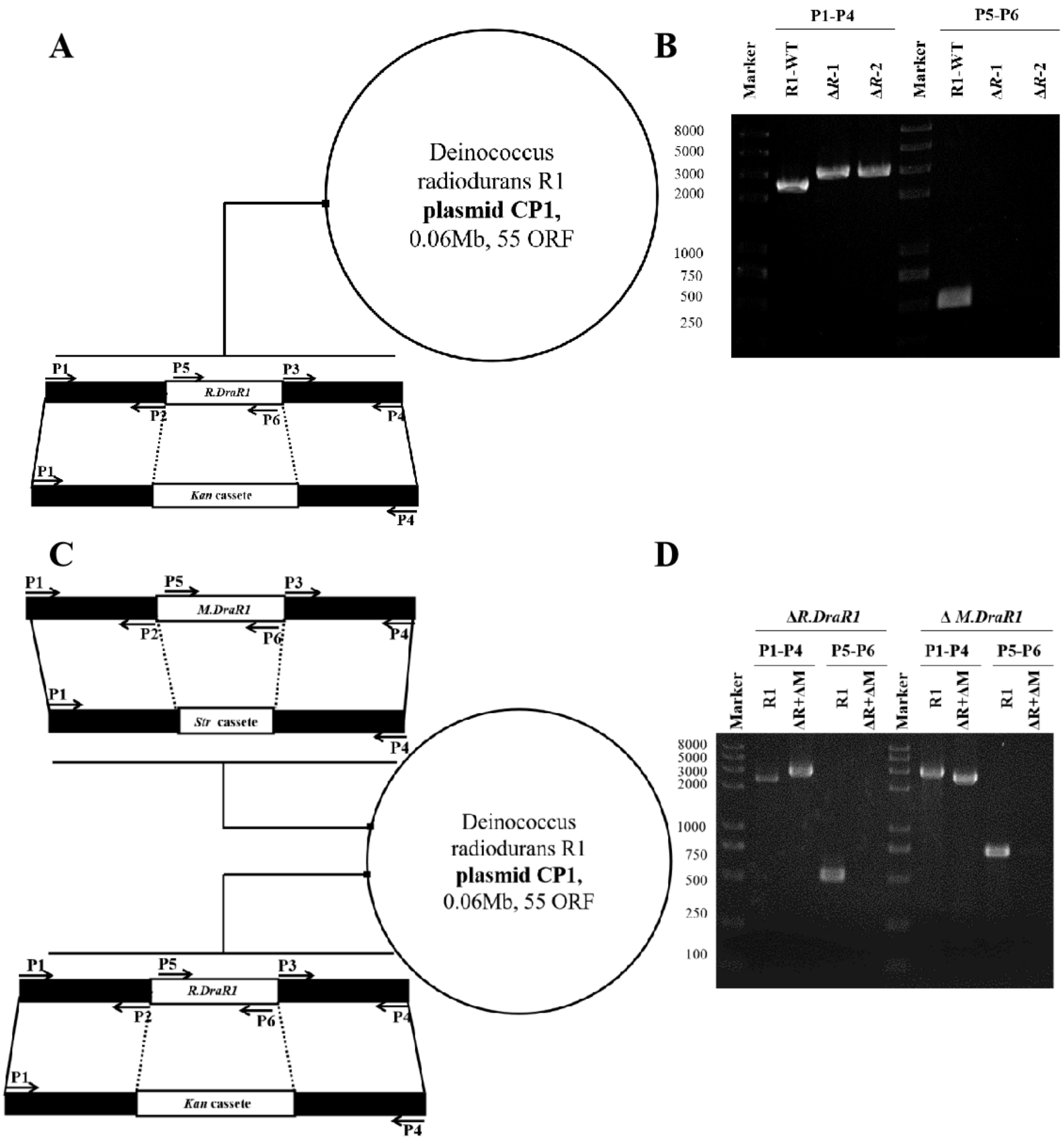

Supplementary Figure S1. Deletion of *R.DraR1* and double deletion of *R.DraR1* and *M.DraR1* gene in *Deinococcus radiodurans* strain. (A,C) Scheme of gene mutation by homologous recombination which replaced *R.DraR1* with kanamycin and *M.DraR1* with streptomycin resistant fragment. (B) PCR analysis to confirm the mutation of *R.DraR1* strain. ΔR-1 and ΔR-2 are 2 clones of mutant. The amplicon from Δ*R.DraR1* (P1-P4,3010 bp) is longer than WT (P1-P4, 2628 bp), and no products to corresponding to the size of the inner fragment from WT (P5-P6, 558 bp) in Δ*R.DraR1,* suggesting *R.DraR1* was completely replaced. (D) PCR analysis to confirm the double mutation of *R.DraR1* and *M.DraR1* strain. The amplicon from Δ*M.DraR1* (P1-P4,2648 bp) is slightly shorter than WT (P1-P4, 3062 bp), and no products to corresponding to the size of the inner fragment from WT (Δ*R.DraR1* P5-P6, 558 bp; Δ*M.DraR1* P5-P6, 706 bp), suggesting 2 genes were completely replaced.

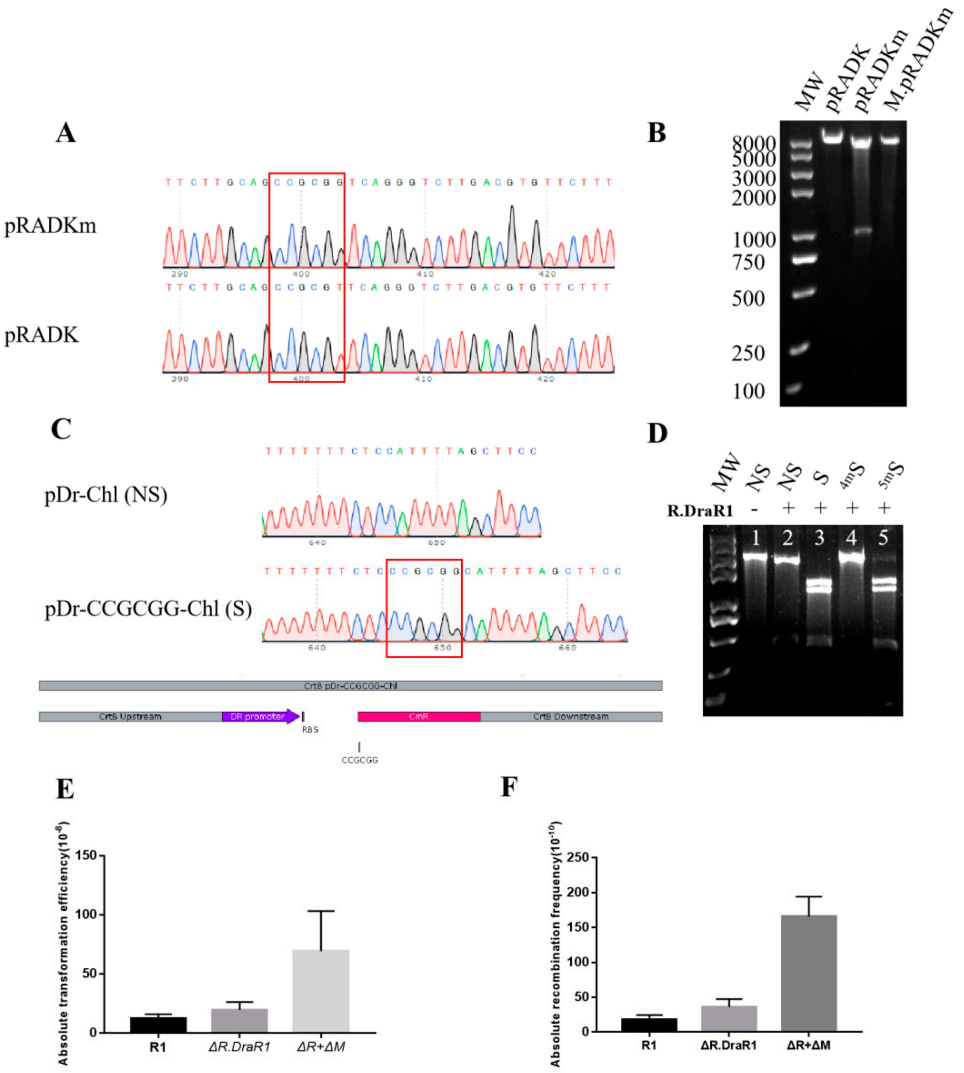

Supplementary Figure S2. Sequencing and methylation and restriction assays to confirm the methylation status in CCGCGG sites. (A) Confirmation of the mutation of T to G to acquire one CCGCGG site for the construction of pRADKm through PCR. pRADK has no CCGCGG site. (B) Confirmation of methylation status of different plasmids. Plasmids were generated using the methylation-deficient *E. coli* ER2796 cells. Acquisition of M.pRADKm follows protection Assays protocol, which involves reacting M.DraR1 under buffer 1 conditions at 30°C for 2 hours, followed by reagent recovery and purification using the kit. The fact that pRADKm can be digested by R.DraR1 but pRADK and M.pRADKm cannot, indicates that all CCGCGG sites in M.pRADKm are methylated. (C) Confirmation of the addition of the CCGCGG Sequence. NS represents fragments that have 1 site, while Each of S fragments have 2 sites. Confirmation of methylation status of different fragments. All fragments were inserted into the pUC19 plasmid and transformed into ER2796. The NS fragment itself contains a CCGCGG site upstream of chloromycetin coding region, which can be cleaved by R.DraR1 (lane 2). The unmethylated S fragment can be cleaved by R.DraR1 (lane 3), while the cleavage activity is inhibited by 4mC M.DraR1 (lane 4), but not by 5mC M.SssI (lane 5). (E) The absolute transformation efficiency of *R1*, *ΔR.DraR1* and *ΔR+ΔM*. (F) The absolute recombination efficiency of *R1*, *ΔR.DraR1* and *ΔR+ΔM*.

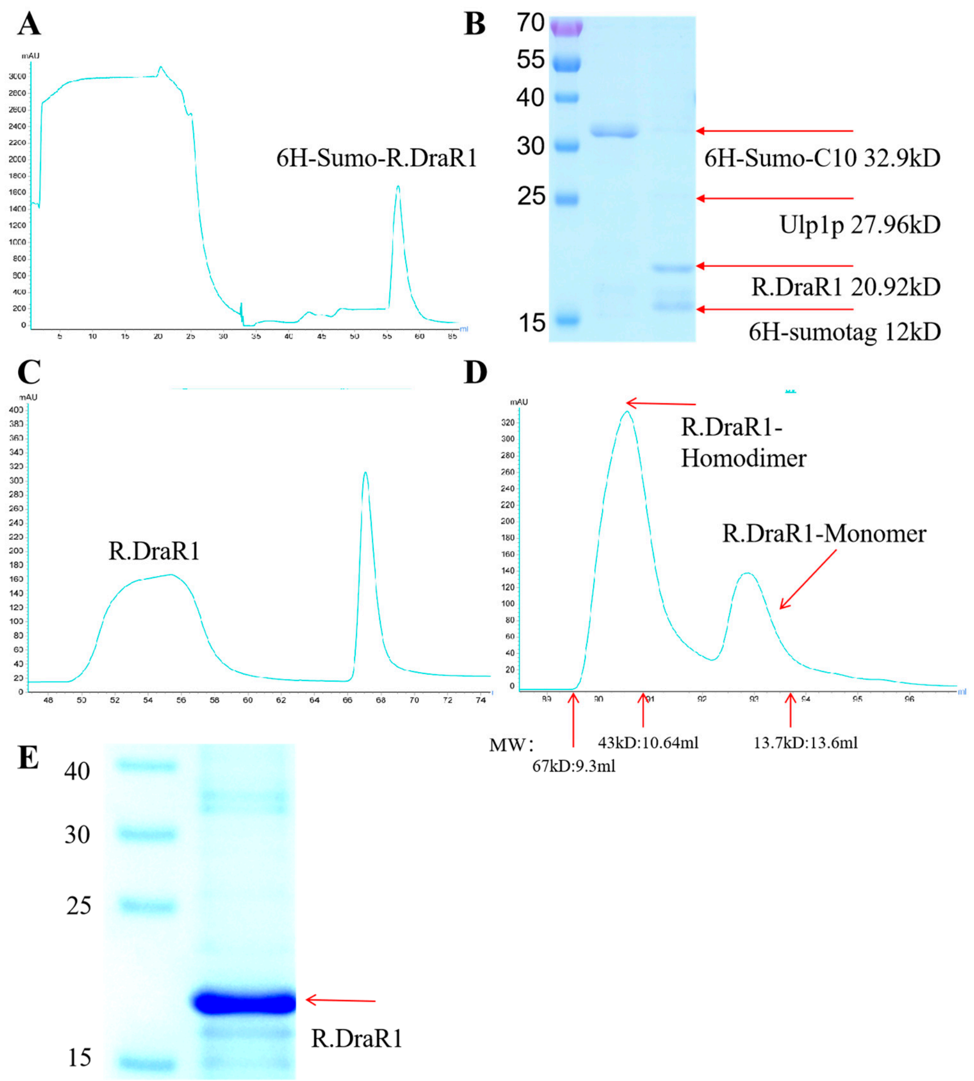

Supplementary Figure S3. Purification and identification of R.DraR1 enzyme. (A) Diagrams of protein purification by HisTrap. The fused 6His-Sumo-R.DraR1 protein was eluted with 500 mM imidazole. (B) SDS-PAGE was used to show that the fusion protein was cleaved by Sumotag protease Ulp1p. (C) Diagrams of protein purification by HisTrap. After Ulp1 protease cleaved overnight at 4 °C, The protein was desalted and loaded onto HisTrap again, the flow-through fractions were collected. (D) Gel-filtration analysis revealed that R.DraR1 exist as a homodimer in solution. FPLC system coupled to a Superdex 75 10/300 GL column. The First peak was collected, R.DraR1 was concentrated to 20 μM, and stored at -20 °C. (E) SDS-PAGE was used to show the fractions collected in (D). The purity of R.DraR1 protein is calculated to be > 90% using ImageJ software. ImageJ software is commonly employed for grayscale analysis to quantify protein purity. To quantify the gel image, it is imported into ImageJ, and the "Analyze > Gels > Select First Lane" command is utilized to designate the lane of interest. Subsequently, the "Analyze > Gels > Plot Lane" command is employed to conduct grayscale quantification. After obtaining the grayscale signal value through grayscale analysis, protein purity was determined by calculating the ratio of the intensity of the target protein band to the total intensity of the lane.

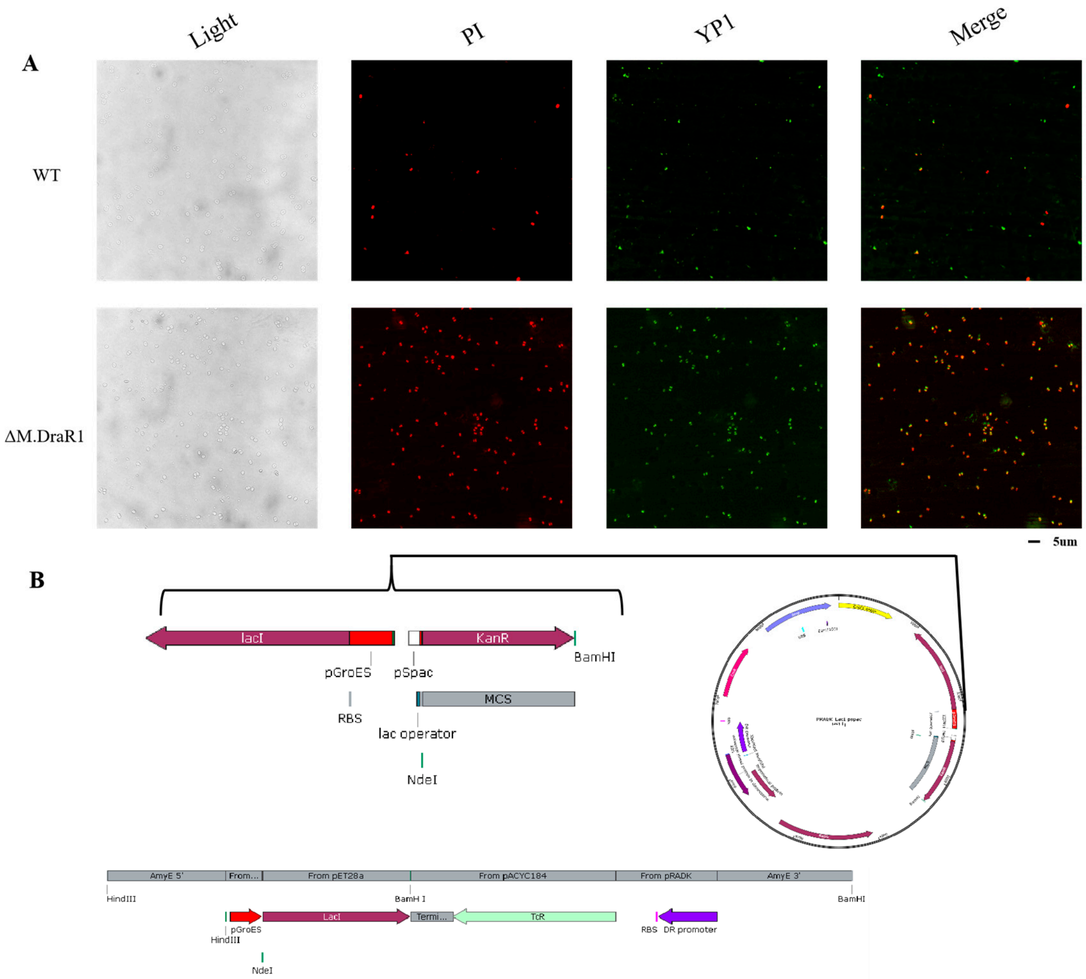

Supplementary Figure S4. Cell Morphology of *D. radiodurans* and construction of IPTG-LacI in *D. radiodurans*. (A) Cell Morphology of *D. radiodurans*. Apoptotic cells are labeled with a green stain, necrotic cells with a red stain, and dead cells with both colors. (B) Construction of IPTG-induced plasmid pRADKIS and the pGroES-LacI-pDr-tetR fragment. The fragment is used for inserting it into the AmyE locus and introducing LacI into the genome of D. radiodurans. Briefly, first we recombine the pGroES-LacI-pDr-tetR fragment into the D. radiodurans AmyE locus and select using tetracycline. Then, we transform the IPTG-induced plasmid pRADKIS into the knockout strain and select using chloromycetin to confirm successful construction.
